# Supplementary material for: Gene dosage imbalance disrupts systemic metabolism in the Dp16 Down syndrome mouse model
Source: bioRxiv. 2026 Jan 14:2026.01.13.699318. Preprint. [Version 2] doi: 10.64898/2026.01.13.699318 (PMC12871095; doi:10.64898/2026.01.13.699318)

bioRxiv preprint doi: https://doi.org/10.64898/2026.01.13.699318; this version posted January 14, 2026. The copyright holder for this preprint (which was not certified by peer review) is the author/funder, who has granted bioRxiv a license to display the preprint in perpetuity. It is made available under aCC-BY 4.0 International license.

SUPPLEMENTAL FIGURE FILES LEGENDS AND SOURCE DATA

Figure 2 – figure supplement 1. Body and tissue weights of chow-fed male and female mice at termination of study. Tissues were collected from chow-fed male and female mice at 27.5 weeks of age. Body weights and the absolute (A and C) and relative (B and D; % of body weight) weights of gWAT, iWAT, liver, and kidney in Dp16 and WT male (A-B) and female (C-D) mice. gWAT, gonadal white adipose tissue; iWAT, inguinal white adipose tissue. Sample size: WT male = 10; Dp16 male = 30; WT female = 15; Dp16 female = 10. All data are presented as mean ± SEM. * P<0.05; ** P<0.01; *** P<0.001.

Figure 2 – figure supplement 2. ANCOVA analysis of energy expenditure in chow-fed mice where lean mass is used as a covariate. ANCOVA analysis of WT and Dp16 male mice across the circadian cycle (dark and light) in *ad libitum* fed (A), fasted (B), and refed (C) states. ANCOVA analysis of WT and Dp16 female mice across the circadian cycle (dark and light) in *ad libitum* fed (D), fasted (E), and refed (F) states. Male Sample size: WT = 12; Dp16 = 12. Female sample size: WT = 15; Dp16 = 6.

Figure 2 – figure supplement 3. Reduced mitochondrial activity in the brown adipose tissue (BAT) of Dp16 mice. Mitochondrial respiration through complex I (CI), CII, and CIV in BAT of WT and Dp16 male and female mice fed a standard chow. **(A and D)** Average oxygen consumption rate (OCR) traces per group, normalized to mitochondrial content. Each group tracing represents the average trace of 10 WT and 9-10 Dp16 samples. Each tracing shows the entire process of the Seahorse-based respirometry assay with injection compounds listed at the time of introduction to the sample. The sequence is as follows: i) basal reads, ii) addition of NADH (activation of respiration through complex I), iii) addition of antimycin A (AA, inhibitor of complex III) and rotenone (Rot, inhibitor of complex I), iv) addition of TMPD and ascorbate (to activate complex IV via electron donation to cytochrome c), and finally v) addition of azide (inhibitor of complex IV). **(B and E)** The same information as presented in (A and D) conducted on the same samples, but the NADH injection step is replaced with the injection of succinate (to activate respiration through complex II) and rotenone (to inhibit complex I). **(C and F)** Average values of all data presented for BAT. Each data point represents the average of three technical replicates measured at three separate times. Both independent measurements of complex IV (CIV) were used to determine average CIV respiration. **** $p < 0.0001$ (two-way ANOVA with Sidak's multiple comparison).

Figure 2 – figure supplement 4. Serum Triiodothyronine (T3), sex and stress hormone levels in WT and Dp16 mice fed a standard chow. (A) Serum T3 levels in male and female mice. (B) Serum testosterone levels in male mice. (C) Serum estradiol levels in female mice. (D) Serum corticosterone in male and female mice. Sample size: male WT = 8-10; male Dp16 = 25-30; female WT = 14-15; female Dp16 = 10.

Figure 3 – figure supplement 1. Liver triacylglycerol (TAG), diacylglycerol (DAG), and cholesterol levels in chow-fed Dp16 mice. Quantification of hepatic TAG and DAG (by TLC method), and cholesterol (by infinity assay kit) levels in chow-fed Dp16 male (A-C) and female mice (D-F) and their corresponding WT controls. Sample size: male WT = 10 and Dp16 = 30; female WT = 15 and Dp16 = 10.

Figure 3 – figure supplement 2. Mitochondrial activity in the liver of Dp16 mice. Mitochondrial respiration through complex I (CI), CII, and CIV in the liver of WT and Dp16 male and female mice fed a standard chow. **(A and D)** Average oxygen consumption rate (OCR) traces per group, normalized to mitochondrial content. Each group tracing represents the average trace of 10 WT and 10 Dp16 samples. Each tracing shows the entire process of the Seahorse-based respirometry assay with injection compounds listed at the time of introduction to the sample. The sequence is as follows: i) basal reads, ii) addition of NADH (activation of respiration through complex I), iii) addition of antimycin A (AA, inhibitor of

bioRxiv preprint doi: https://doi.org/10.64898/2026.01.13.699318; this version posted January 14, 2026. The copyright holder for this preprint (which was not certified by peer review) is the author/funder, who has granted bioRxiv a license to display the preprint in perpetuity. It is made available under aCC-BY 4.0 International license.

complex III) and rotenone (Rot, inhibitor of complex I), iv) addition of TMPD and ascorbate (to activate complex IV via electron donation to cytochrome c), and finally v) addition of azide (inhibitor of complex IV). **(B and E)** The same information as presented in (A and D) conducted on the same samples, but the NADH injection step is replaced with the injection of succinate (to activate respiration through complex II) and rotenone (to inhibit complex I). **(C and F)** Average values of all data presented for liver. Each data point represents the average of three technical replicates measured at three separate times. Both independent measurements of complex IV (CIV) were used to determine average CIV respiration.

Figure 4 – Source data 1. Supplemental table of differential metabolites in Dp16 male mouse liver vs WT controls. Differential metabolites criteria: VIP > 1.0,   fold change (FC) > 1.2 or FC < 0.833 and *P*-value < 0.05. Sample name notation: male WT liver (M_WT_L), male WT serum (M_WT_S), male Dp16 liver (M_16_L), male Dp16 serum (M_16_S), female WT liver (F_WT_L), female WT serum (F_WT_L), female Dp16 liver (F_16_L), female Dp16 serum (F_16_S).

Figure 4 – Source data 2. Supplemental table of differential metabolites in Dp16 female mouse liver vs WT controls. Differential metabolites criteria: VIP > 1.0,   fold change (FC) > 1.2 or FC < 0.833 and *P*-value < 0.05. Sample name notation: male WT liver (M_WT_L), male WT serum (M_WT_S), male Dp16 liver (M_16_L), male Dp16 serum (M_16_S), female WT liver (F_WT_L), female WT serum (F_WT_L), female Dp16 liver (F_16_L), female Dp16 serum (F_16_S).

Figure 4 – Source data 3. Supplemental table of differential metabolites in Dp16 male mouse serum vs WT controls. Differential metabolites criteria: VIP > 1.0,   fold change (FC) > 1.2 or FC < 0.833 and *P*-value < 0.05. Sample name notation: male WT liver (M_WT_L), male WT serum (M_WT_S), male Dp16 liver (M_16_L), male Dp16 serum (M_16_S), female WT liver (F_WT_L), female WT serum (F_WT_L), female Dp16 liver (F_16_L), female Dp16 serum (F_16_S).

Figure 4 – Source data 4. Supplemental table of differentially expressed metabolites in Dp16 female mouse serum vs WT controls. Differential metabolites criteria: VIP > 1.0,   fold change (FC) > 1.2 or FC < 0.833 and *P*-value < 0.05. Sample name notation: male WT liver (M_WT_L), male WT serum (M_WT_S), male Dp16 liver (M_16_L), male Dp16 serum (M_16_S), female WT liver (F_WT_L), female WT serum (F_WT_L), female Dp16 liver (F_16_L), female Dp16 serum (F_16_S).

Figure 4 – Source data 5. Supplemental table showing the shared and distinct differential metabolites in Dp16 male and female mouse liver and serum vs WT controls. Sample name notation: male WT liver (M_WT_L), male WT serum (M_WT_S), male Dp16 liver (M_16_L), male Dp16 serum (M_16_S), female WT liver (F_WT_L), female WT serum (F_WT_L), female Dp16 liver (F_16_L), female Dp16 serum (F_16_S).

Figure 4 – figure supplement 1. Differential metabolites found in the liver and serum of Dp16 male and female mice. Volcano plots showing differential metabolites up- and down-regulated in Dp16 male liver (A), female liver (B), male serum (C), and female serum (D). *n* = 6 per genotype. VIP, Variable Importance in Projection. VIP scores provide a quantitative measure of a metabolite's discriminatory power between different groups. Metabolites with a VIP score of 1.0 or greater are considered significant.

Figure 4 – figure supplement 2. KEGG classification analysis of liver metabolites. KEGG classification plots based on the differential metabolites from male (A) and female (B) Dp16 mouse liver vs WT control. The horizontal coordinates in the graph indicate the number of metabolites annotated under a particular KEGG pathway as a percentage of the number of all annotated metabolites, the vertical coordinates are KEGG pathway primary classifications on the right and KEGG pathway secondary classifications on the left.

bioRxiv preprint doi: https://doi.org/10.64898/2026.01.13.699318; this version posted January 14, 2026. The copyright holder for this preprint (which was not certified by peer review) is the author/funder, who has granted bioRxiv a license to display the preprint in perpetuity. It is made available under aCC-BY 4.0 International license.

Figure 4 – figure supplement 3. KEGG classification analysis of serum metabolites. KEGG classification plots based on the differential metabolites from male (A) and female (B) Dp16 mouse liver vs WT control. The horizontal coordinates in the graph indicate the number of metabolites annotated under a particular KEGG pathway as a percentage of the number of all annotated metabolites, the vertical coordinates are KEGG pathway primary classifications on the right and KEGG pathway secondary classifications on the left.

Figure 4 – figure supplement 4. Serum alanine aminotransferase (ALT) levels in WT and Dp16 mice fed a standard chow. Serum ALT levels in male and female mice. Sample size: male WT = 10; male Dp16 = 27; female WT = 14; female Dp16 = 10.

Figure 5 – Source data 1. Supplemental table with differentially expressed genes (DEGs) upregulated in the gonadal white adipose tissue (gWAT) of chow-fed Dp16 male mice relative to WT controls.

Figure 5 – Source data 2. Supplemental table with differentially expressed genes (DEGs) down-regulated in the gonadal white adipose tissue (gWAT) of chow-fed Dp16 male mice relative to WT controls.

Figure 5 – Source data 3. Supplemental table with differentially expressed genes (DEGs) upregulated in the inguinal white adipose tissue (iWAT) of chow-fed Dp16 male mice relative to WT controls.

Figure 5 – Source data 4. Supplemental table with differentially expressed genes (DEGs) down-regulated in the inguinal white adipose tissue (iWAT) of chow-fed Dp16 male mice relative to WT controls.

Figure 5 – Source data 5. Supplemental table with differentially expressed genes (DEGs) upregulated in the brown adipose tissue (BAT) of chow-fed Dp16 male mice relative to WT controls.

Figure 5 – Source data 6. Supplemental table with differentially expressed genes (DEGs) down-regulated in the brown adipose tissue (BAT) of chow-fed Dp16 male mice relative to WT controls.

Figure 5 – Source data 7. Supplemental table with differentially expressed genes (DEGs) upregulated in the liver of chow-fed Dp16 male mice relative to WT controls.

Figure 5 – Source data 8. Supplemental table with differentially expressed genes (DEGs) down-regulated in the liver of chow-fed Dp16 male mice relative to WT controls.

Figure 5 – Source data 9. Supplemental table with differentially expressed genes (DEGs) upregulated in the skeletal muscle (gastrocnemius) of chow-fed Dp16 male mice relative to WT controls.

Figure 5 – Source data 10. Supplemental table with differentially expressed genes (DEGs) down-regulated in the skeletal muscle (gastrocnemius) of chow-fed Dp16 male mice relative to WT controls.

Figure 5 – Source data 11. Supplemental table with differentially expressed genes (DEGs) upregulated in the hypothalamus of chow-fed Dp16 male mice relative to WT controls.

Figure 5 – Source data 12. Supplemental table with differentially expressed genes (DEGs) down-regulated in the hypothalamus of chow-fed Dp16 male mice relative to WT controls.

bioRxiv preprint doi: https://doi.org/10.64898/2026.01.13.699318; this version posted January 14, 2026. The copyright holder for this preprint (which was not certified by peer review) is the author/funder, who has granted bioRxiv a license to display the preprint in perpetuity. It is made available under aCC-BY 4.0 International license.

Figure 5 – Source data 13. Supplemental table with differentially expressed genes (DEGs) upregulated in the gonadal white adipose tissue (gWAT) of chow-fed Dp16 female mice relative to WT controls.

Figure 5 – Source data 14. Supplemental table with differentially expressed genes (DEGs) down-regulated in the gonadal white adipose tissue (gWAT) of chow-fed Dp16 female mice relative to WT controls.

Figure 5 – Source data 15. Supplemental table with differentially expressed genes (DEGs) upregulated in the inguinal white adipose tissue (iWAT) of chow-fed Dp16 female mice relative to WT controls.

Figure 5 – Source data 16. Supplemental table with differentially expressed genes (DEGs) down-regulated in the inguinal white adipose tissue (iWAT) of chow-fed Dp16 female mice relative to WT controls.

Figure 5 – Source data 17. Supplemental table with differentially expressed genes (DEGs) upregulated in the brown adipose tissue (BAT) of chow-fed Dp16 female mice relative to WT controls.

Figure 5 – Source data 18. Supplemental table with differentially expressed genes (DEGs) down-regulated in the brown adipose tissue (BAT) of chow-fed Dp16 female mice relative to WT controls.

Figure 5 – Source data 19. Supplemental table with differentially expressed genes (DEGs) upregulated in the liver of chow-fed Dp16 female mice relative to WT controls.

Figure 5 – Source data 20. Supplemental table with differentially expressed genes (DEGs) down-regulated in the liver of chow-fed Dp16 female mice relative to WT controls.

Figure 5 – Source data 21. Supplemental table with differentially expressed genes (DEGs) upregulated in the skeletal muscle (gastrocnemius) of chow-fed Dp16 female mice relative to WT controls.

Figure 5 – Source data 22. Supplemental table with differentially expressed genes (DEGs) down-regulated in the skeletal muscle (gastrocnemius) of chow-fed Dp16 female mice relative to WT controls.

Figure 5 – Source data 23. Supplemental table with differentially expressed genes (DEGs) upregulated in the hypothalamus of chow-fed Dp16 female mice relative to WT controls.

Figure 5 – Source data 24. Supplemental table with differentially expressed genes (DEGs) down-regulated in the hypothalamus of chow-fed Dp16 female mice relative to WT controls.

Figure 5 – figure supplement 1. Differentially expressed genes (DEGs) involved in ER stress, fibrosis, glucose and lipid metabolism that are up- or down-regulated in the inguinal white adipose tissue (iWAT) of Dp16 mice.

Figure 5 – figure supplement 2. Differentially expressed genes (DEGs) involved in immune activation, lipid metabolism, and mitochondrial respiration that are up- or down-regulated in the brown adipose tissue (BAT) of Dp16 mice.

Figure 5 – figure supplement 3. Differentially expressed genes (DEGs) involved in immune activation, lipid metabolism, and mitochondrial respiration that are up- or down-regulated in the liver of Dp16 mice.

bioRxiv preprint doi: https://doi.org/10.64898/2026.01.13.699318; this version posted January 14, 2026. The copyright holder for this preprint (which was not certified by peer review) is the author/funder, who has granted bioRxiv a license to display the preprint in perpetuity. It is made available under aCC-BY 4.0 International license.

Figure 5 – figure supplement 4. Differentially expressed genes (DEGs) involved in immune response, metabolism, mitochondrial respiration, and Wnt signaling that are up- or down-regulated in the skeletal muscle (gastrocnemius) of Dp16 mice.

Figure 5 – figure supplement 5. Differentially expressed genes (DEGs) involved in immune response and extracellular matrix that are upregulated in the hypothalamus of Dp16 mice.

Figure 5 – figure supplement 6. Hydroxyproline (marker of fibrosis) and malondialdehyde (marker of oxidative stress) levels in the liver, gWAT, and iWAT of chow-fed Dp16 mice. (A-F) Quantification of hydroxyproline content in the liver, gWAT, and iWAT of Dp16 male (A-C) and female (D-F) mice and their corresponding WT controls. gWAT, gonadal white adipose tissue; iWAT, inguinal white adipose tissue. Sample size: male WT = 7-10 and Dp16 = 27-29; female WT = 10-13 and Dp16 = 7-10. **(G-L)** Quantification of malondialdehyde (MDA) levels in the liver, gWAT, and iWAT of Dp16 male (G-I) and female (J-L) mice and their corresponding WT controls. gWAT, gonadal white adipose tissue; iWAT, inguinal white adipose tissue. Sample size: male WT = 6-10 and Dp16 = 25-30; female WT = 8-13 and Dp16 = 6-10. All data are presented as mean ± SEM. * $P<0.05$; ** $P<0.01$

Figure 6 – figure supplement 1. ANCOVA analysis of energy expenditure in HFD-fed mice where lean mass is used as a covariate. ANCOVA analysis of WT and Dp16 male mice across the circadian cycle (dark and light) in *ad libitum* fed (A), fasted (B), and refed (C) states. ANCOVA analysis of WT and Dp16 female mice across the circadian cycle (dark and light) in *ad libitum* fed (D), fasted (E), and refed (F) states. Male Sample size: WT = 12; Dp16 = 12. Female sample size: WT = 14; Dp16 = 14.

Figure 6 – figure supplement 2. Serum Triiodothyronine (T3), sex and stress hormone levels in WT and Dp16 mice fed a high-fat diet. (A) Serum T3 levels in male and female mice. (B) Serum testosterone levels in male mice. (C) Serum estradiol levels in female mice. (D) Serum corticosterone in male and female mice. Sample size: male WT = 9-13; male Dp16 = 11-12; female WT = 14-15; female Dp16 = 14.

Figure 6 – figure supplement 3. Body and tissue weights of HFD-fed male and female mice at termination of study. Tissues were collected from male mice (50 weeks old) after they had been fed a high-fat diet for 34.5 weeks. Body weights and the absolute (A) and relative (B; % of body weight) weights of gWAT, iWAT, liver, and kidney in Dp16 and WT male mice. Female tissues (45 weeks old) were mice had been fed a high-fat diet for 26 weeks. Body weights and the absolute (A) and relative (B; % of body weight) weights of gWAT, iWAT, liver, heart, and kidney in Dp16 and WT female mice. gWAT, gonadal white adipose tissue; iWAT, inguinal white adipose tissue. Sample size: WT male = 14; Dp16 male = 12; WT female = 14; Dp16 female = 12. All data are presented as mean ± SEM. * $P<0.05$; ** $P<0.01$; *** $P<0.001$; **** $P<0.0001$.

Figure 6 – figure supplement 4. Hydroxyproline (marker of fibrosis) and nalondialdehyde (marker of oxidative stress) levels in the liver, gWAT, and iWAT of Dp16 mice on HFD. (A-F) Quantification of hydroxyproline content in the liver, gWAT, and iWAT of Dp16 male (A-C) and female (D-F) mice and their corresponding WT controls. gWAT, gonadal white adipose tissue; iWAT, inguinal white adipose tissue. Sample size: male WT = 13-14 and Dp16 = 10-12; female WT = 10-14 and Dp16 = 12-14. **(G-L)** Quantification of malondialdehyde (MDA) levels in the liver, gWAT, and iWAT of Dp16 male (G-I) and female (J-L) mice and their corresponding WT controls. gWAT, gonadal white adipose tissue; iWAT, inguinal white adipose tissue. Sample size: male WT = 13-14 and Dp16 = 12; female WT = 14 and Dp16 = 13-14. All data are presented as mean ± SEM. * $P<0.05$; ** $P<0.01$

bioRxiv preprint doi: https://doi.org/10.64898/2026.01.13.699318; this version posted January 14, 2026. The copyright holder for this preprint (which was not certified by peer review) is the author/funder, who has granted bioRxiv a license to display the preprint in perpetuity. It is made available under aCC-BY 4.0 International license.

Fig. 2 - figure supplement 1

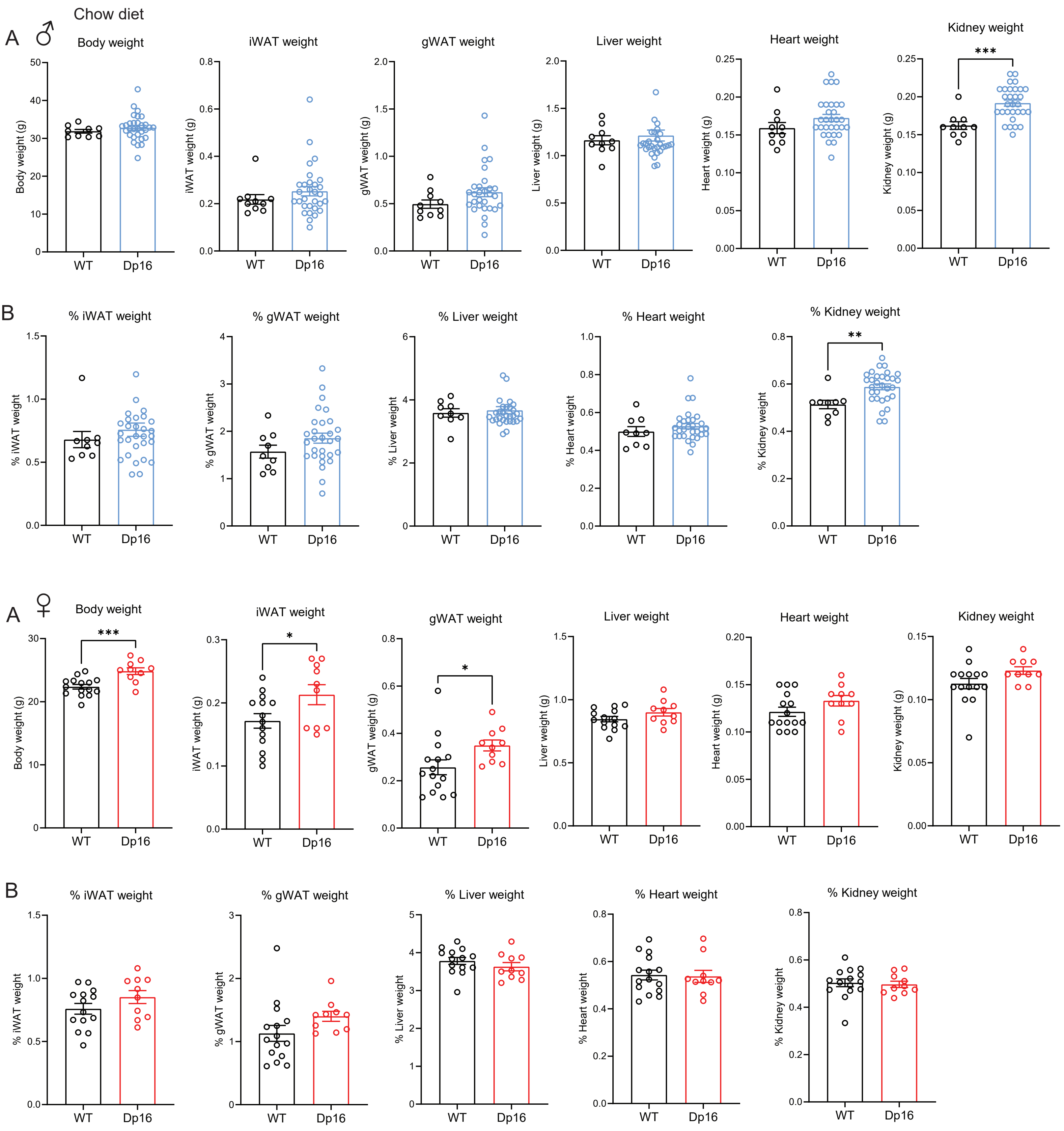

bioRxiv preprint doi: https://doi.org/10.64898/2026.01.13.699318; this version posted January 14, 2026. The copyright holder for this preprint (which was not certified by peer review) is the author/funder, who has granted bioRxiv a license to display the preprint in perpetuity. It is made available under aCC-BY 4.0 International license.

Fig. 2 - figure supplement 2

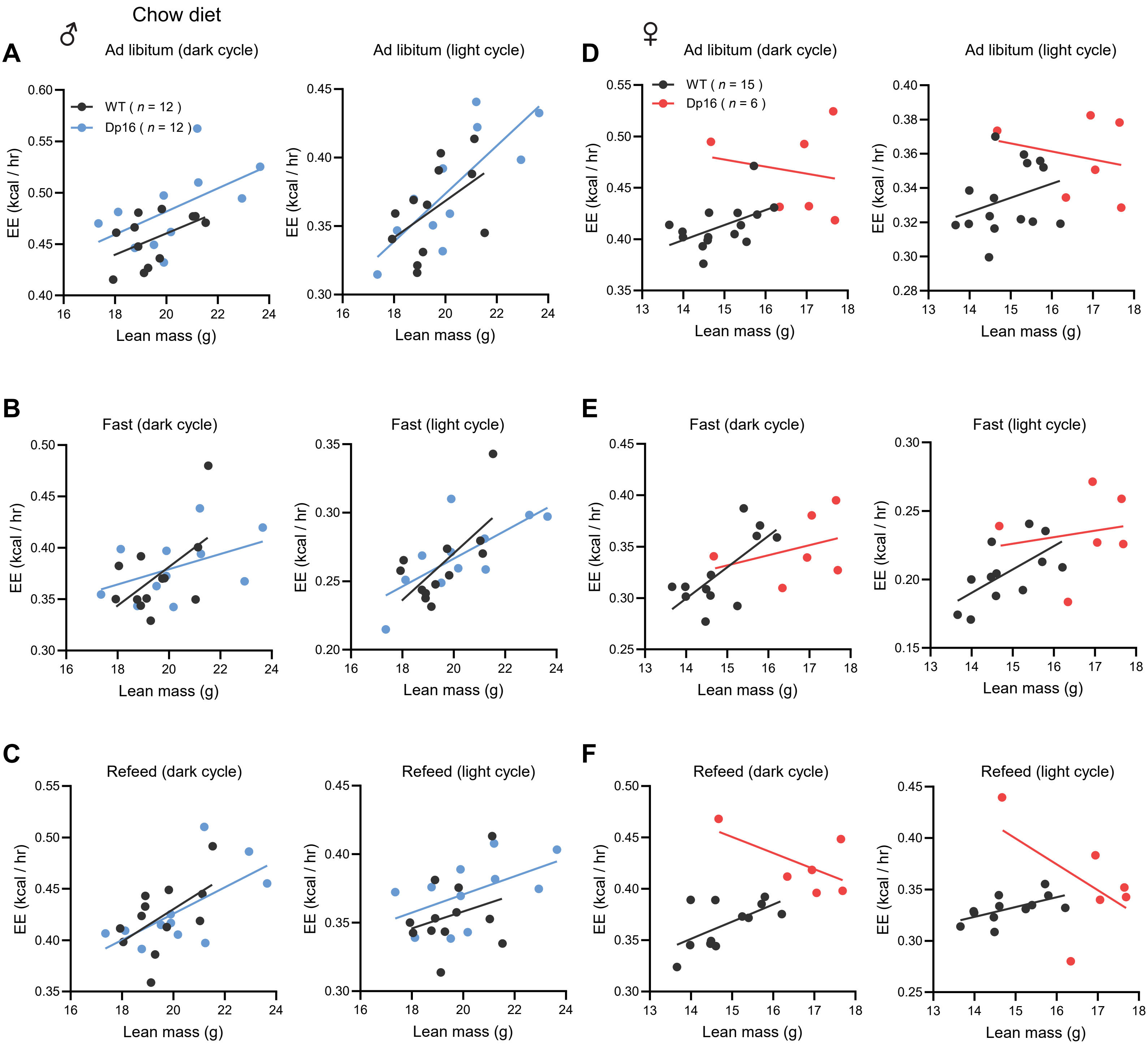

bioRxiv preprint doi: https://doi.org/10.64898/2026.01.13.699318; this version posted January 14, 2026. The copyright holder for this preprint (which was not certified by peer review) is the author/funder, who has granted bioRxiv a license to display the preprint in perpetuity. It is made available under aCC-BY 4.0 International license.

Fig. 2—figure supplement 3

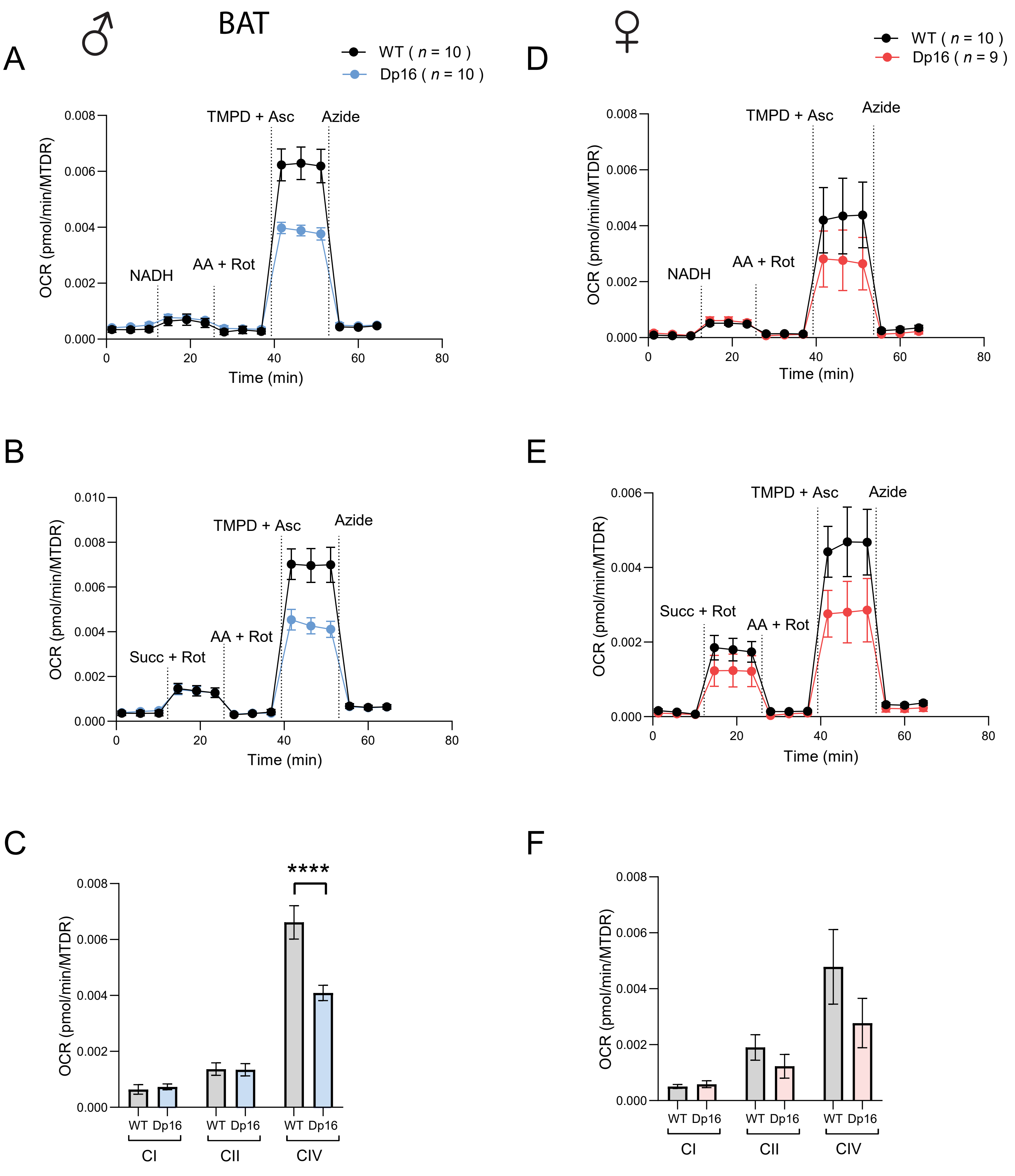

bioRxiv preprint doi: https://doi.org/10.64898/2026.01.13.699318; this version posted January 14, 2026. The copyright holder for this preprint (which was not certified by peer review) is the author/funder, who has granted bioRxiv a license to display the preprint in perpetuity. It is made available under aCC-BY 4.0 International license.

Fig. 2 - figure supplement 4

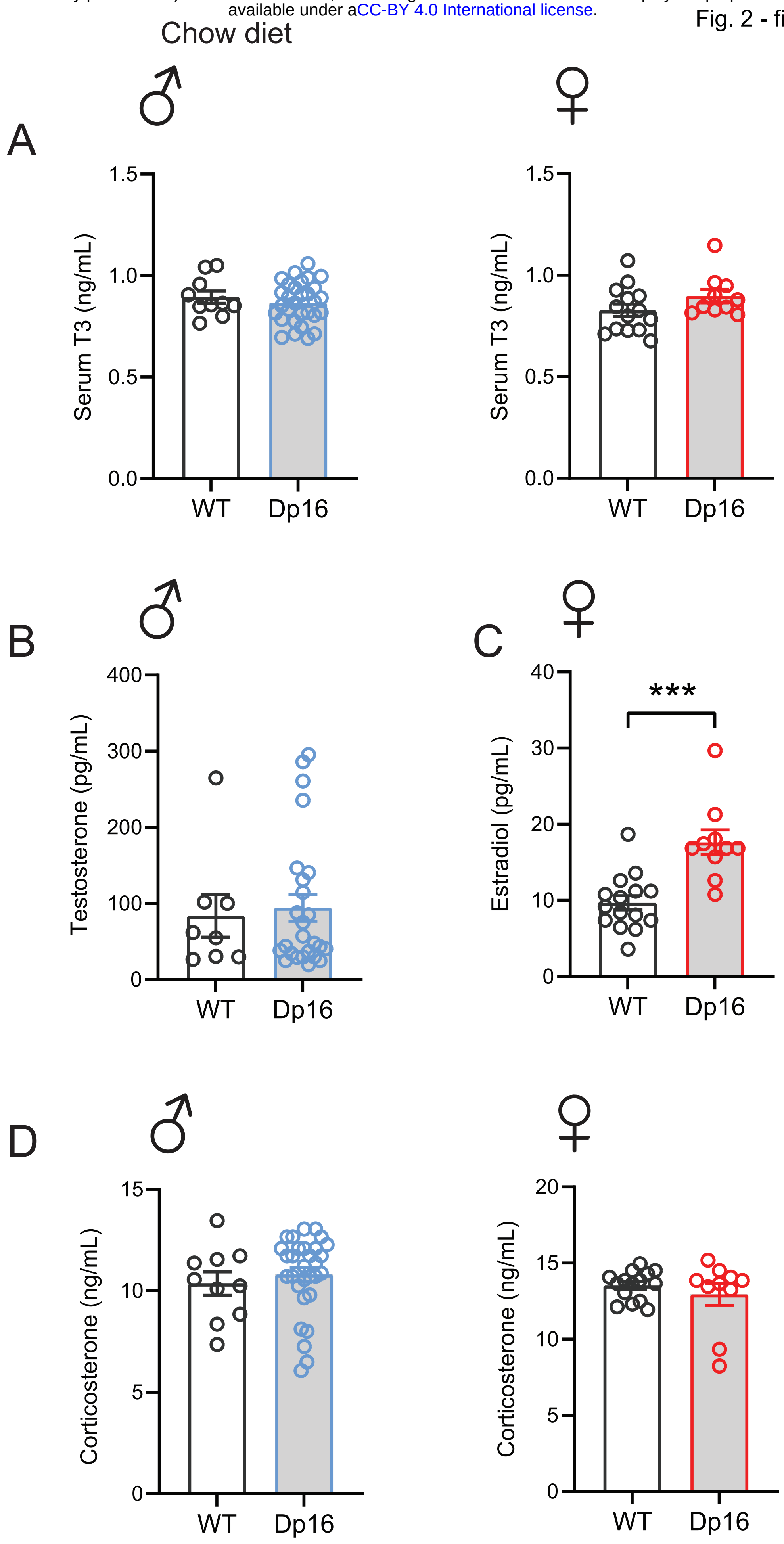

bioRxiv preprint doi: https://doi.org/10.64898/2026.01.13.699318; this version posted January 14, 2026. The copyright holder for this preprint (which was not certified by peer review) is the author/funder, who has granted bioRxiv a license to display the preprint in perpetuity. It is made available under aCC-BY 4.0 International license.

Fig. 3 - figure supplement 1

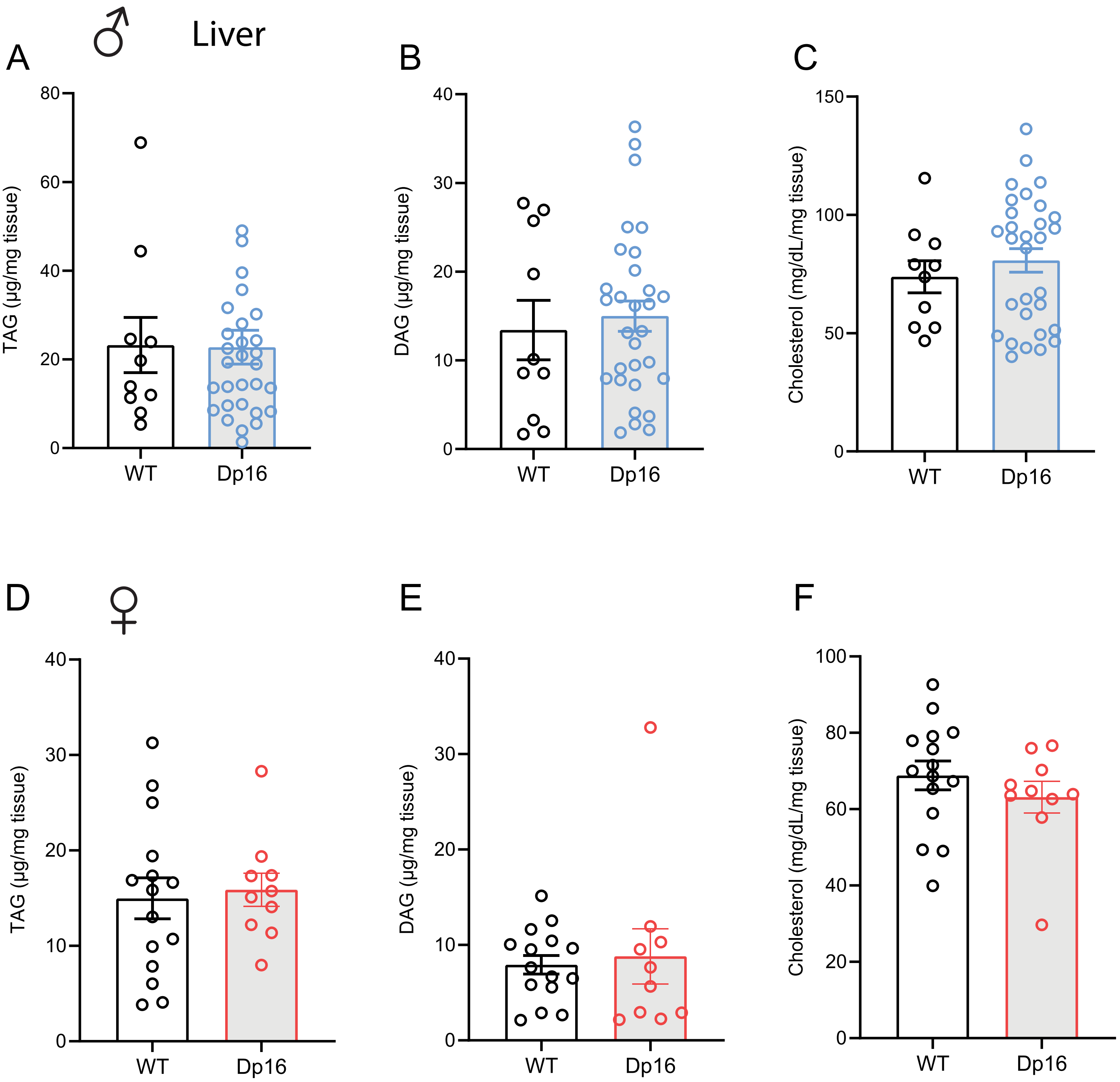

bioRxiv preprint doi: https://doi.org/10.64898/2026.01.13.699318; this version posted January 14, 2026. The copyright holder for this preprint (which was not certified by peer review) is the author/funder, who has granted bioRxiv a license to display the preprint in perpetuity. It is made available under aCC-BY 4.0 International license.

Fig. 3 - figure supplement 2

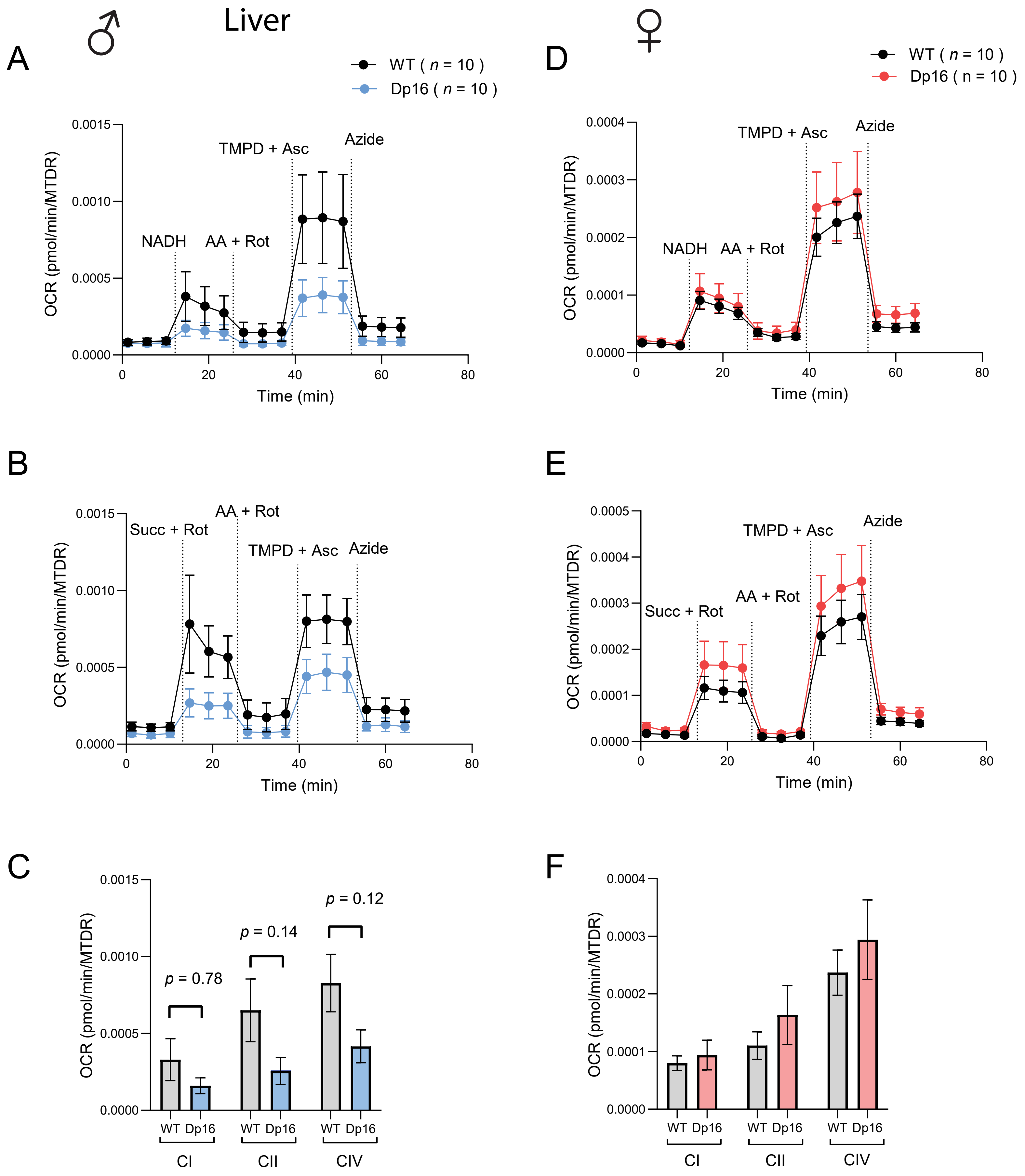

bioRxiv preprint doi: https://doi.org/10.64898/2026.01.13.699318; this version posted January 14, 2026. The copyright holder for this preprint (which was not certified by peer review) is the author/funder, who has granted bioRxiv a license to display the preprint in perpetuity. It is made available under aCC-BY 4.0 International license.

Fig. 4 - figure supplement 1

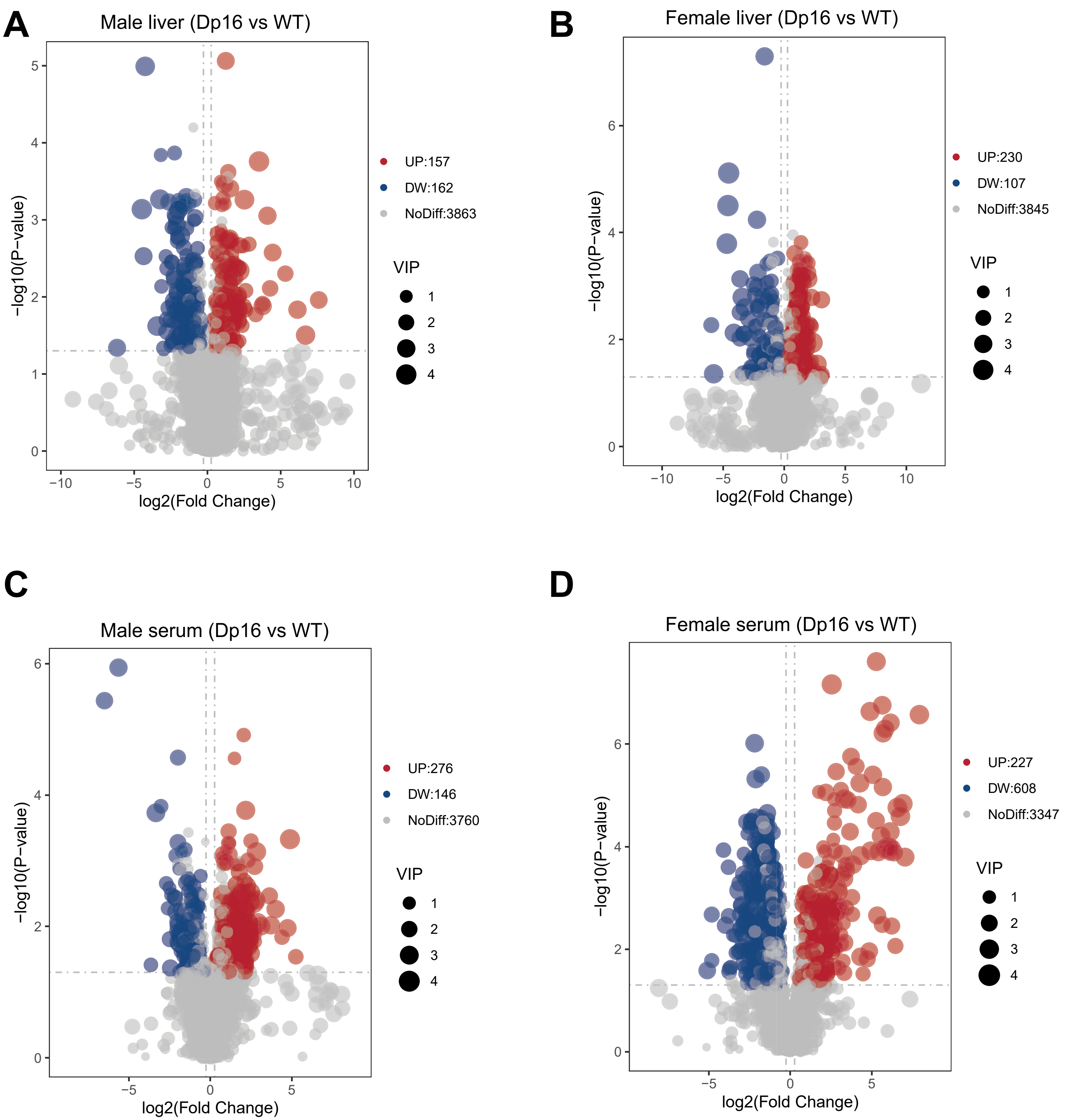

bioRxiv preprint doi: https://doi.org/10.64898/2026.01.13.699318; this version posted January 14, 2026. The copyright holder for this preprint (which was not certified by peer review) is the author/funder, who has granted bioRxiv a license to display the preprint in perpetuity. It is made available under aCC-BY 4.0 International license.

Fig. 4 - figure supplement 2

A Male liver (Dp16 vs WT)

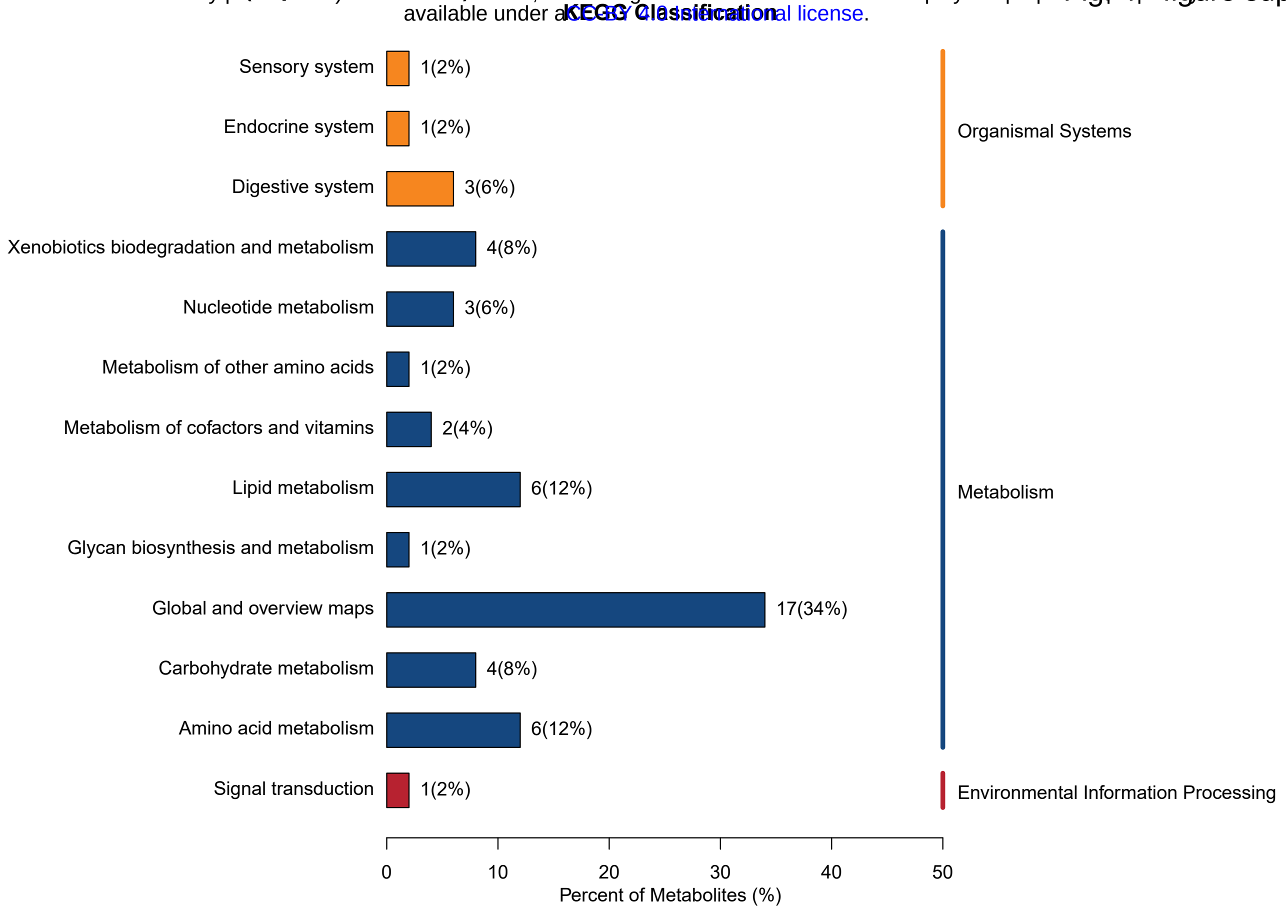

B Female liver (Dp16 vs WT)

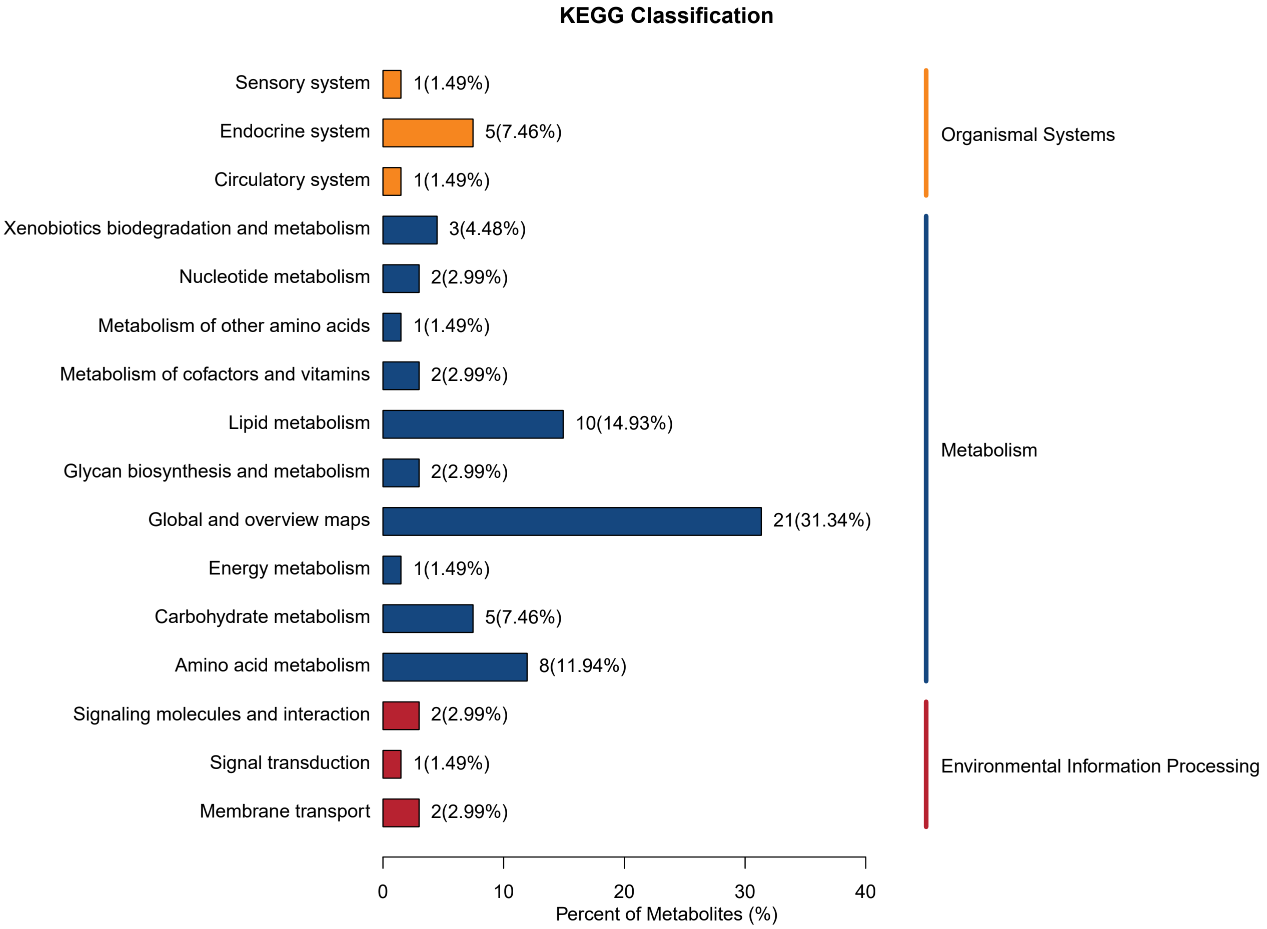

bioRxiv preprint doi: https://doi.org/10.64898/2026.01.13.699318; this version posted January 14, 2026. The copyright holder for this preprint (which was not certified by peer review) is the author/funder, who has granted bioRxiv a license to display the preprint in perpetuity. It is made available under aCC-BY 4.0 International license.

A Male serum (Dp16 vs WT)

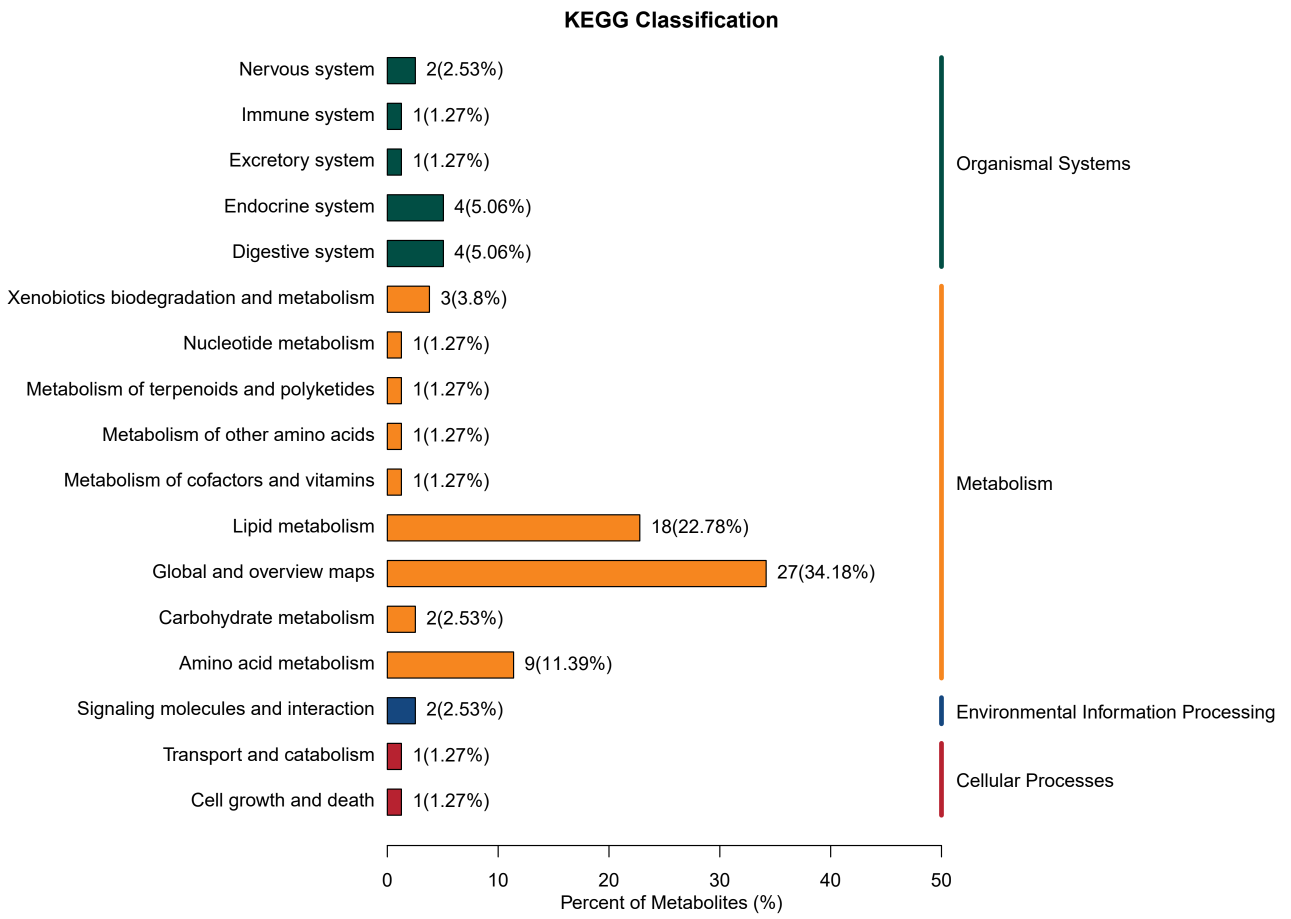

B Female serum (Dp16 vs WT)

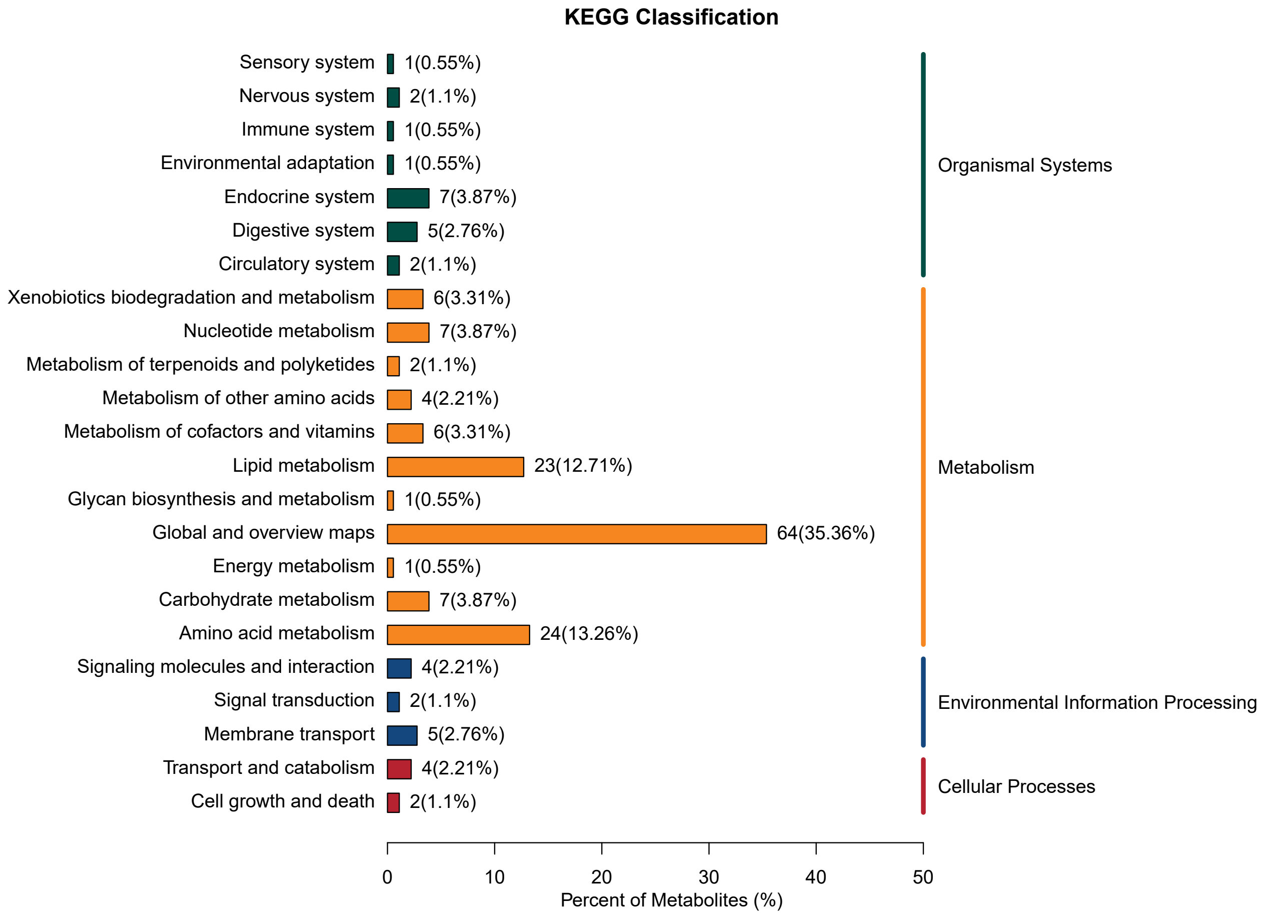

bioRxiv preprint doi: https://doi.org/10.64898/2026.01.13.699318; this version posted January 14, 2026. The copyright holder for this preprint (which was not certified by peer review) is the author/funder, who has granted bioRxiv a license to display the preprint in perpetuity. It is made available under aCC-BY 4.0 International license.

Fig. 4 - figure supplement 4

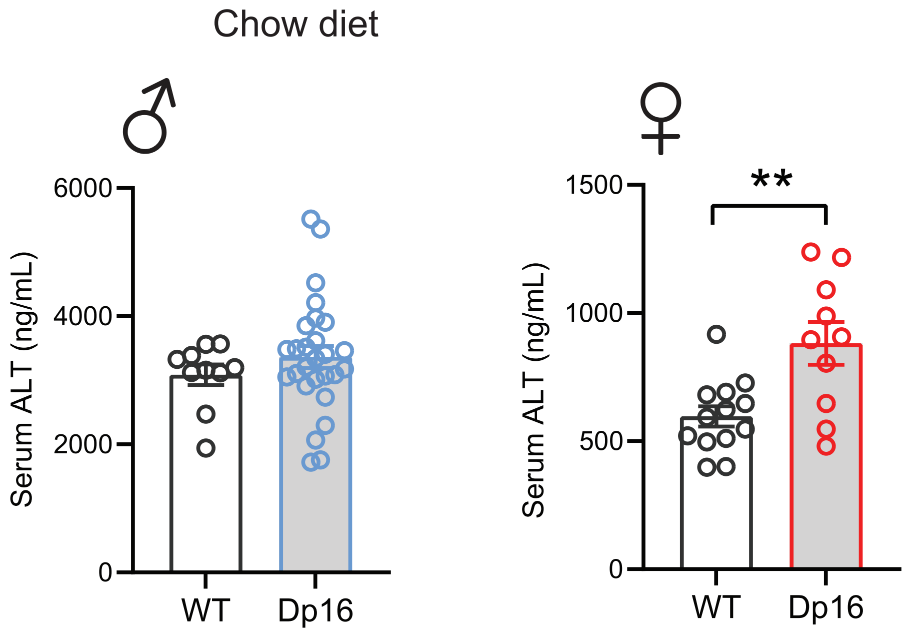

bioRxiv preprint doi: https://doi.org/10.64898/2026.01.13.699318; this version posted January 14, 2026. The copyright holder for this preprint (which was not certified by peer review) is the author/funder, who has granted bioRxiv a license to display the preprint in perpetuity. It is made available under aCC-BY 4.0 International license.

Fig. 5 - figure supplement 1

iWAT

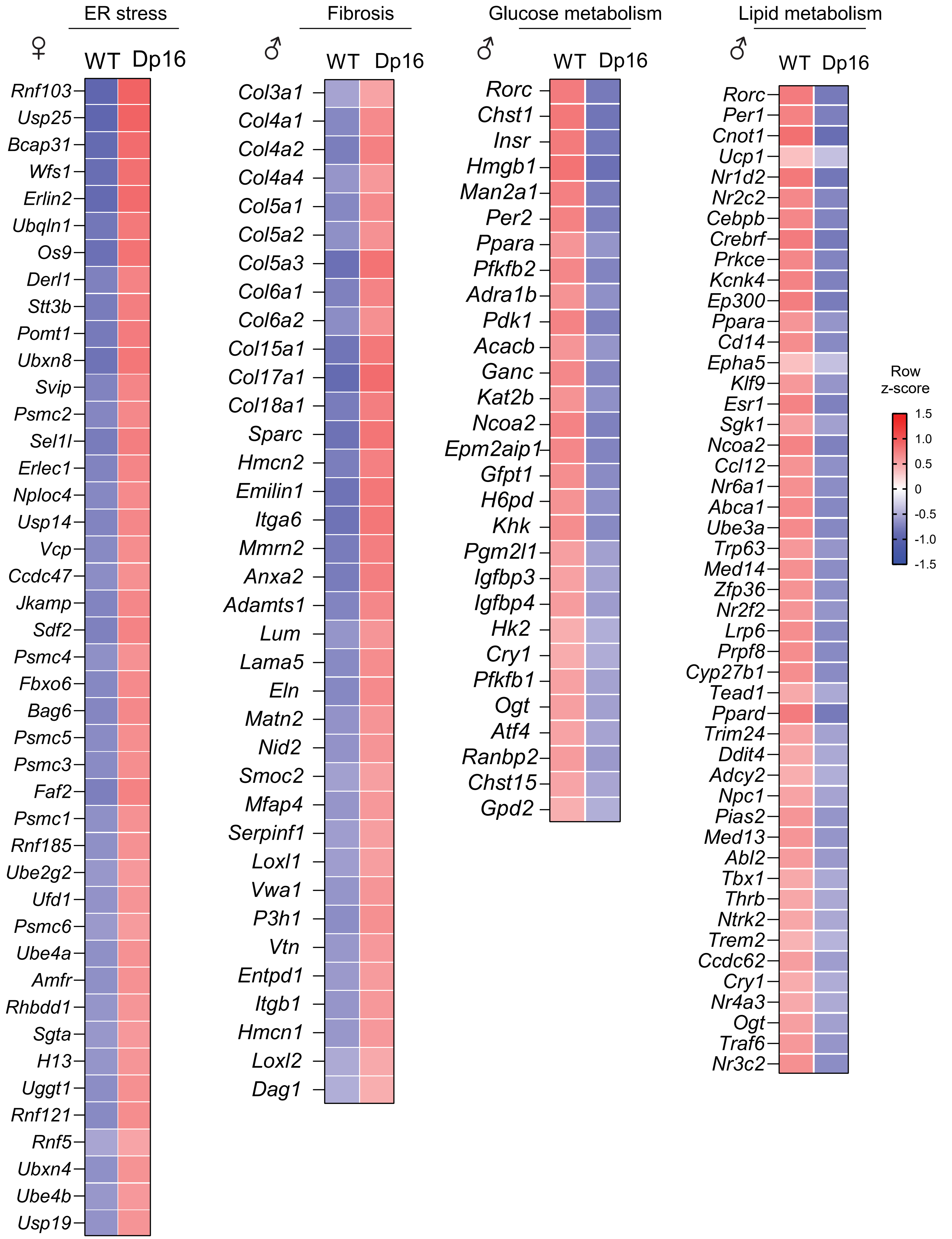

bioRxiv preprint doi: https://doi.org/10.64898/2026.01.13.699318; this version posted January 14, 2026. The copyright holder for this preprint (which was not certified by peer review) is the author/funder, who has granted bioRxiv a license to display the preprint in perpetuity. It is made available under aCC-BY 4.0 International license.

Fig. 5 - figure supplement 2

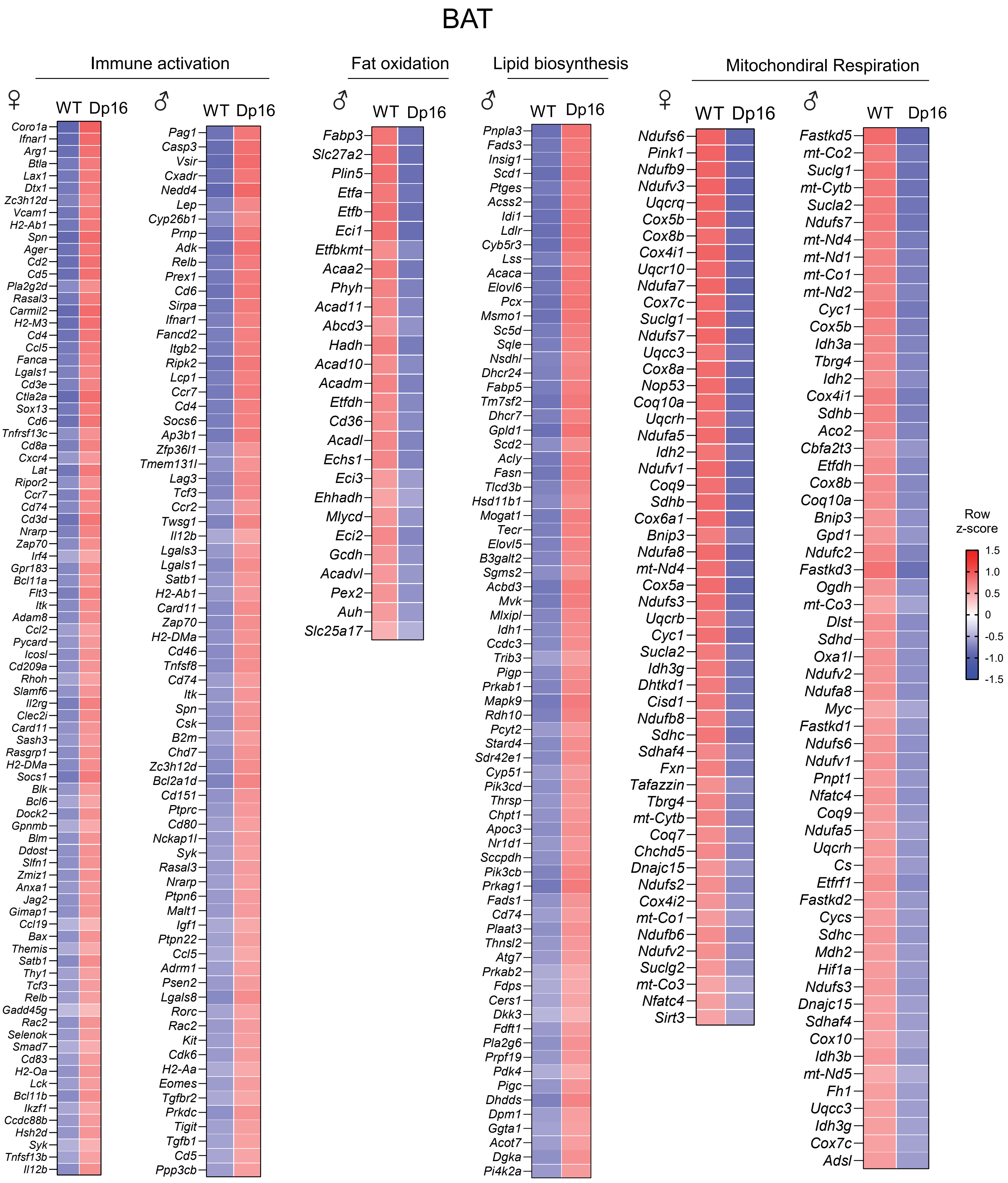

bioRxiv preprint doi: https://doi.org/10.64898/2026.01.13.699318; this version posted January 14, 2026. The copyright holder for this preprint (which was not certified by peer review) is the author/funder, who has granted bioRxiv a license to display the preprint in perpetuity. It is made available under aCC-BY 4.0 International license.

Fig.5—figure supplement 3

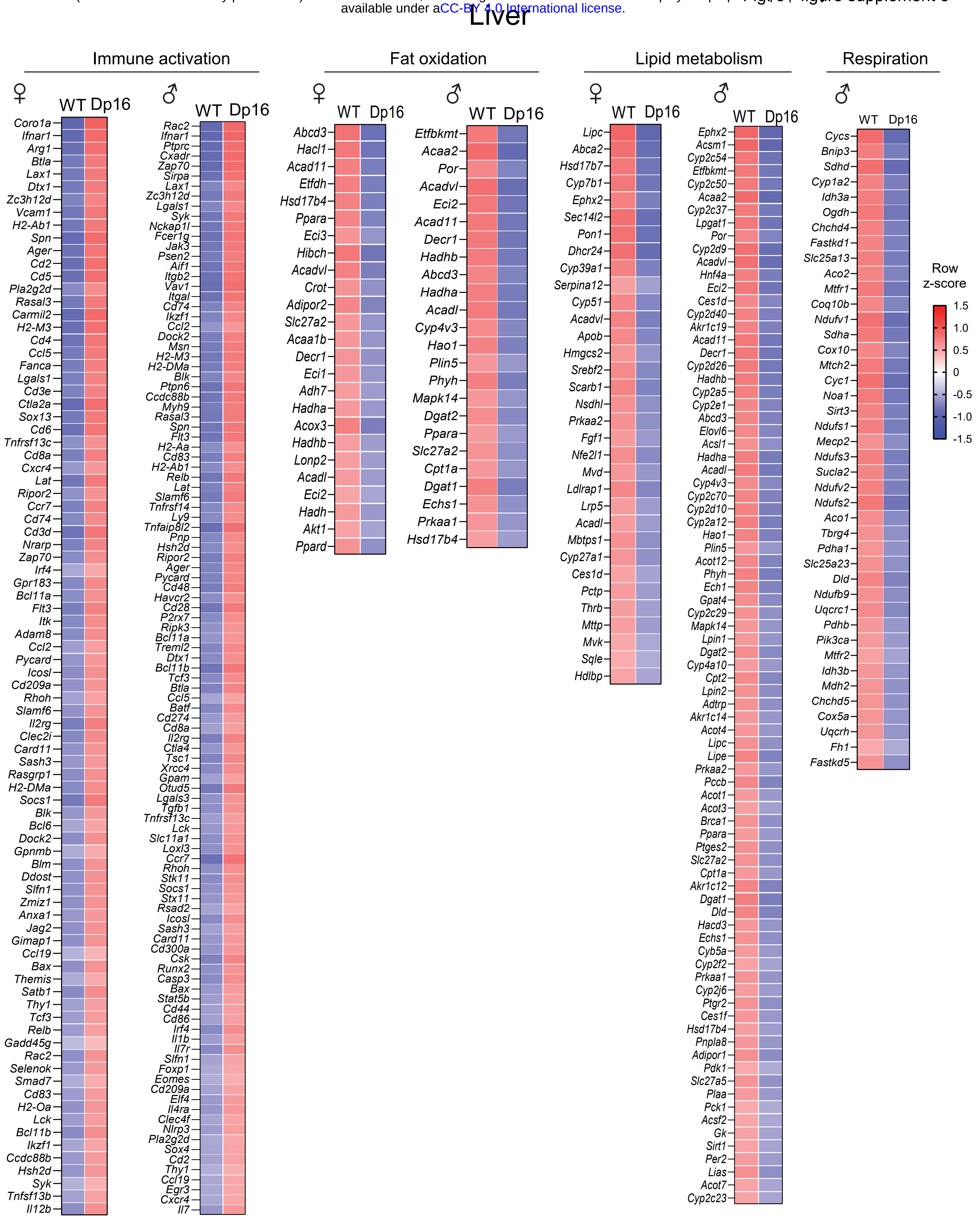

bioRxiv preprint doi: https://doi.org/10.64898/2026.01.13.699318; this version posted January 14, 2026. The copyright holder for this preprint (which was not certified by peer review) is the author/funder, who has granted bioRxiv a license to display the preprint in perpetuity. It is made available under aCC-BY 4.0 International license.

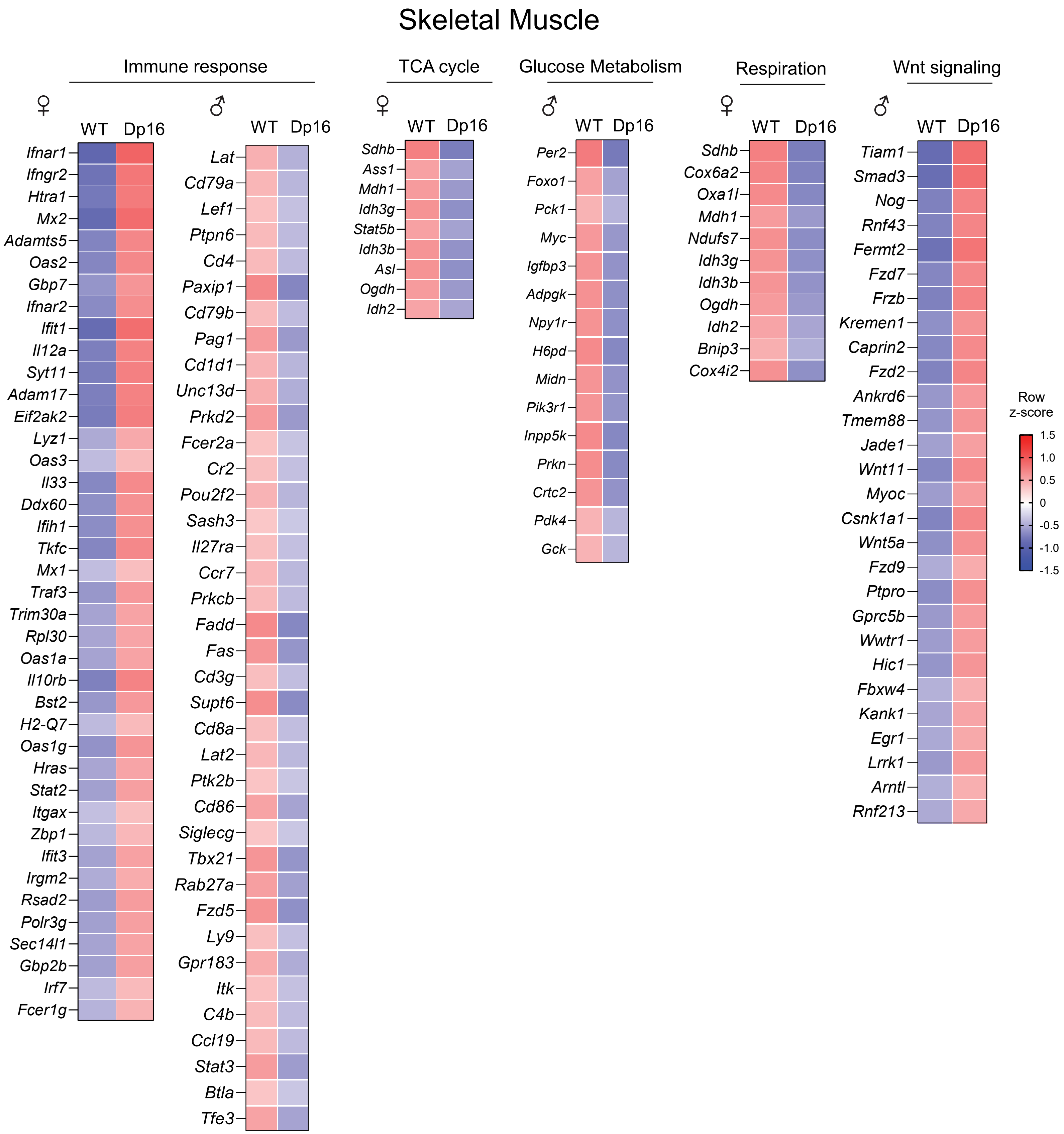

bioRxiv preprint doi: https://doi.org/10.64898/2026.01.13.699318; this version posted January 14, 2026. The copyright holder for this preprint (which was not certified by peer review) is the author/funder, who has granted bioRxiv a license to display the preprint in perpetuity. It is made available under aCC-BY 4.0 International license.

Fig. 5 - figure supplement 5

Hypothalamus

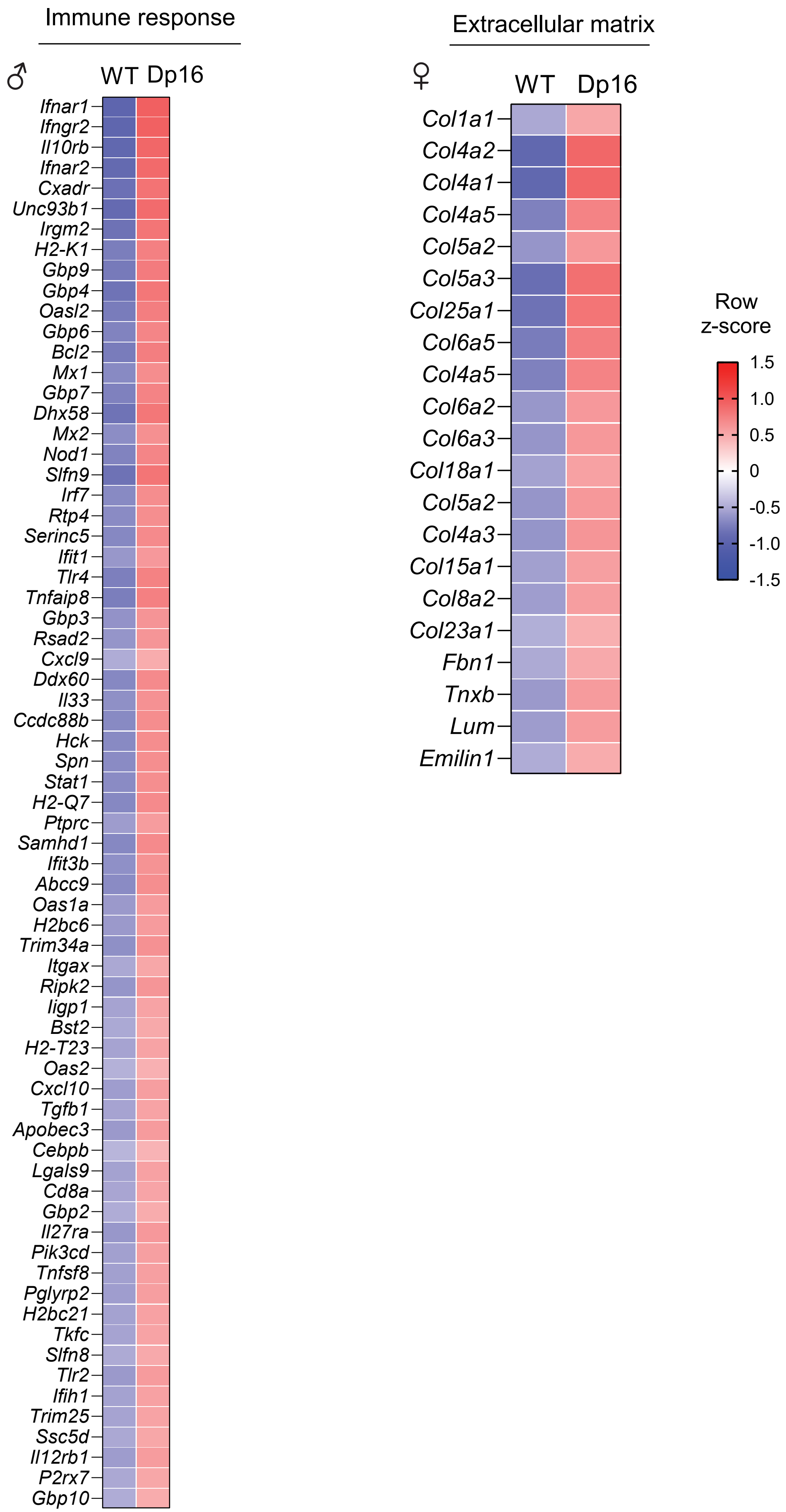

bioRxiv preprint doi: https://doi.org/10.64898/2026.01.13.699318; this version posted January 14, 2026. The copyright holder for this preprint (which was not certified by peer review) is the author/funder, who has granted bioRxiv a license to display the preprint in perpetuity. It is made available under aCC-BY 4.0 International license.

Fig 5 - figure supplement 6

Chow diet (fibrosis)

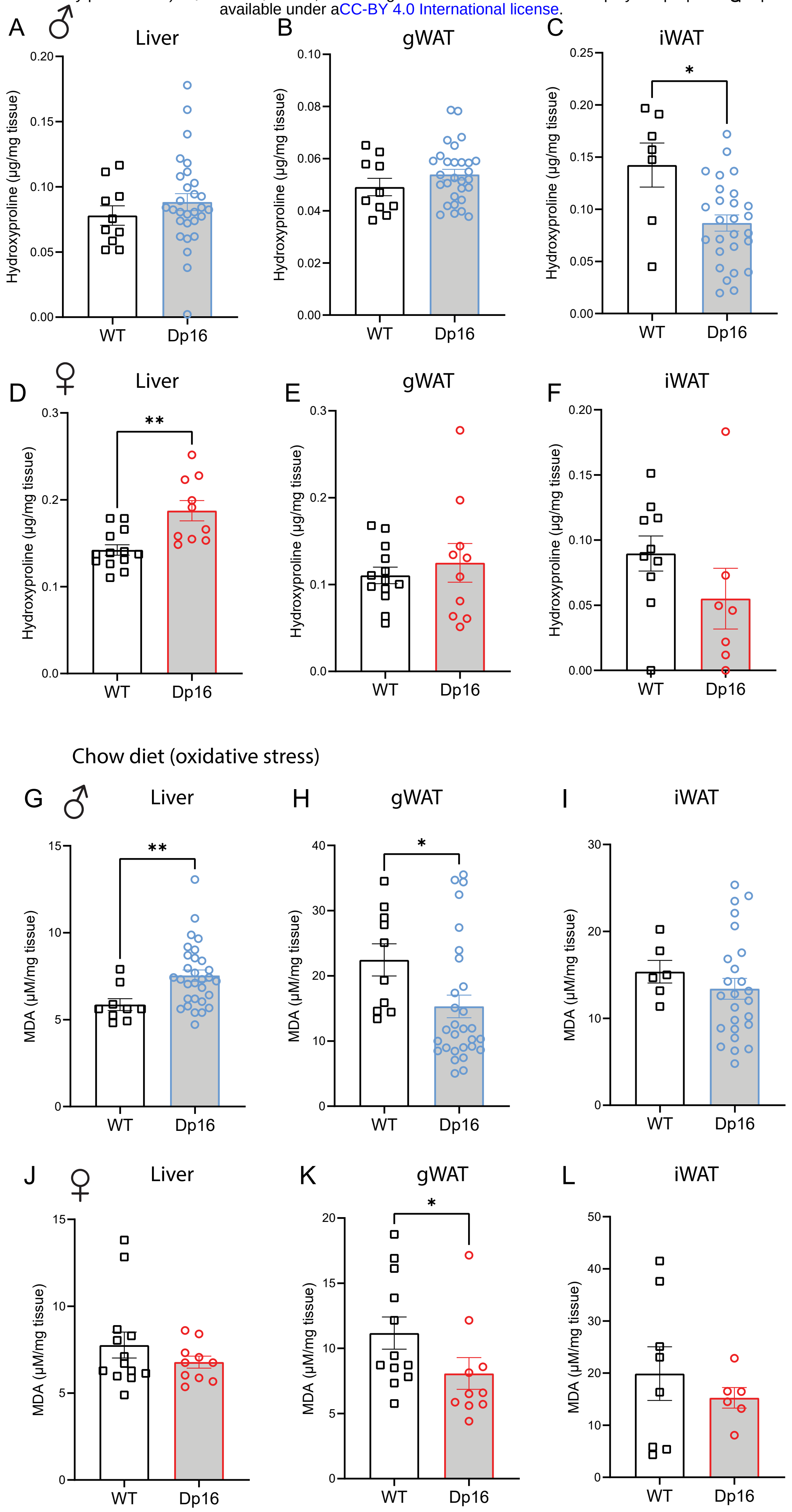

bioRxiv preprint doi: https://doi.org/10.64898/2026.01.13.699318; this version posted January 14, 2026. The copyright holder for this preprint (which was not certified by peer review) is the author/funder, who has granted bioRxiv a license to display the preprint in perpetuity. It is made available under aCC-BY 4.0 International license.

Fig. 6 - figure supplement 1

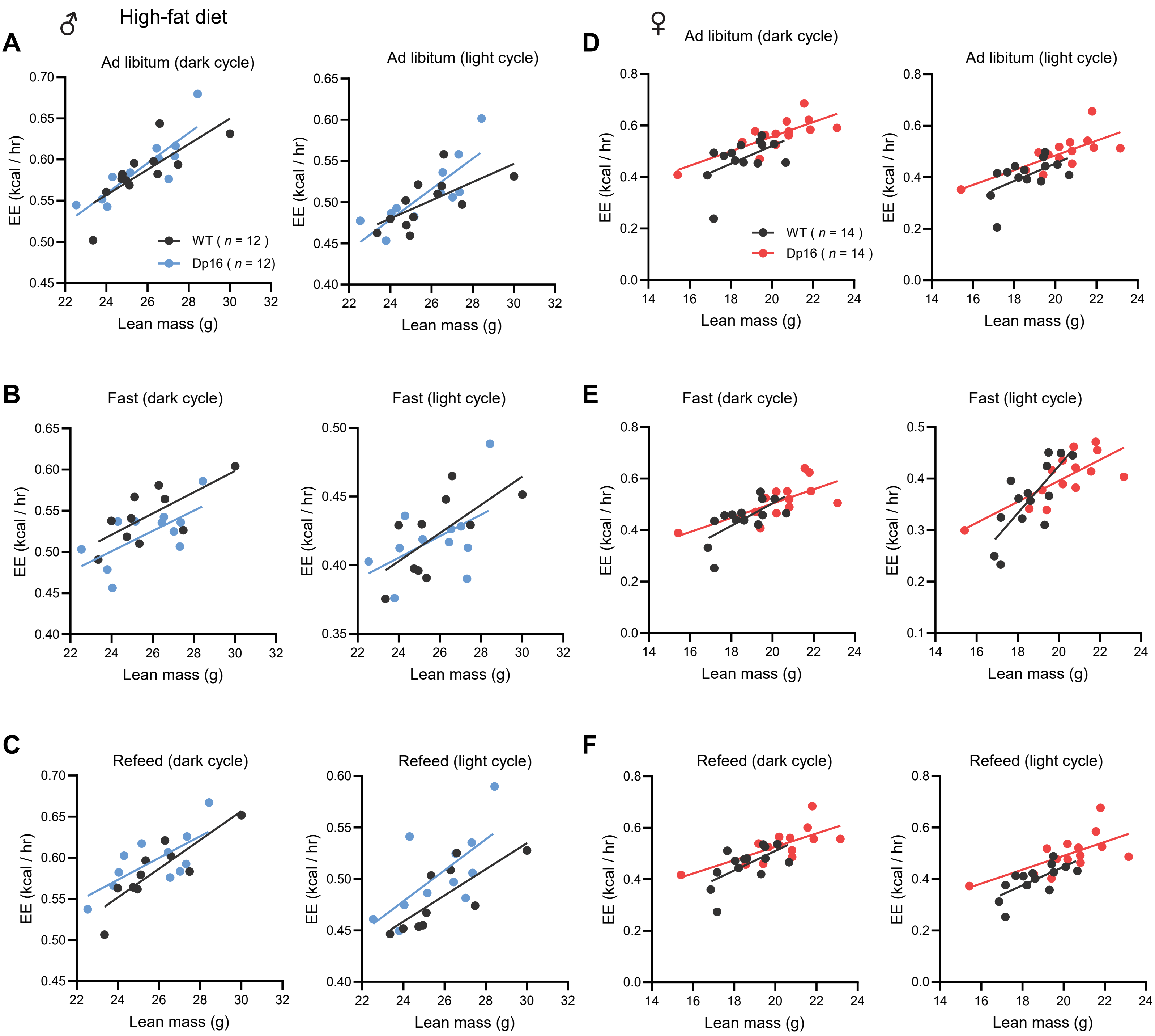

bioRxiv preprint doi: https://doi.org/10.64898/2026.01.13.699318; this version posted January 14, 2026. The copyright holder for this preprint (which was not certified by peer review) is the author/funder, who has granted bioRxiv a license to display the preprint in perpetuity. It is made available under aCC-BY 4.0 International license.

Fig. 6 - figure supplement 2

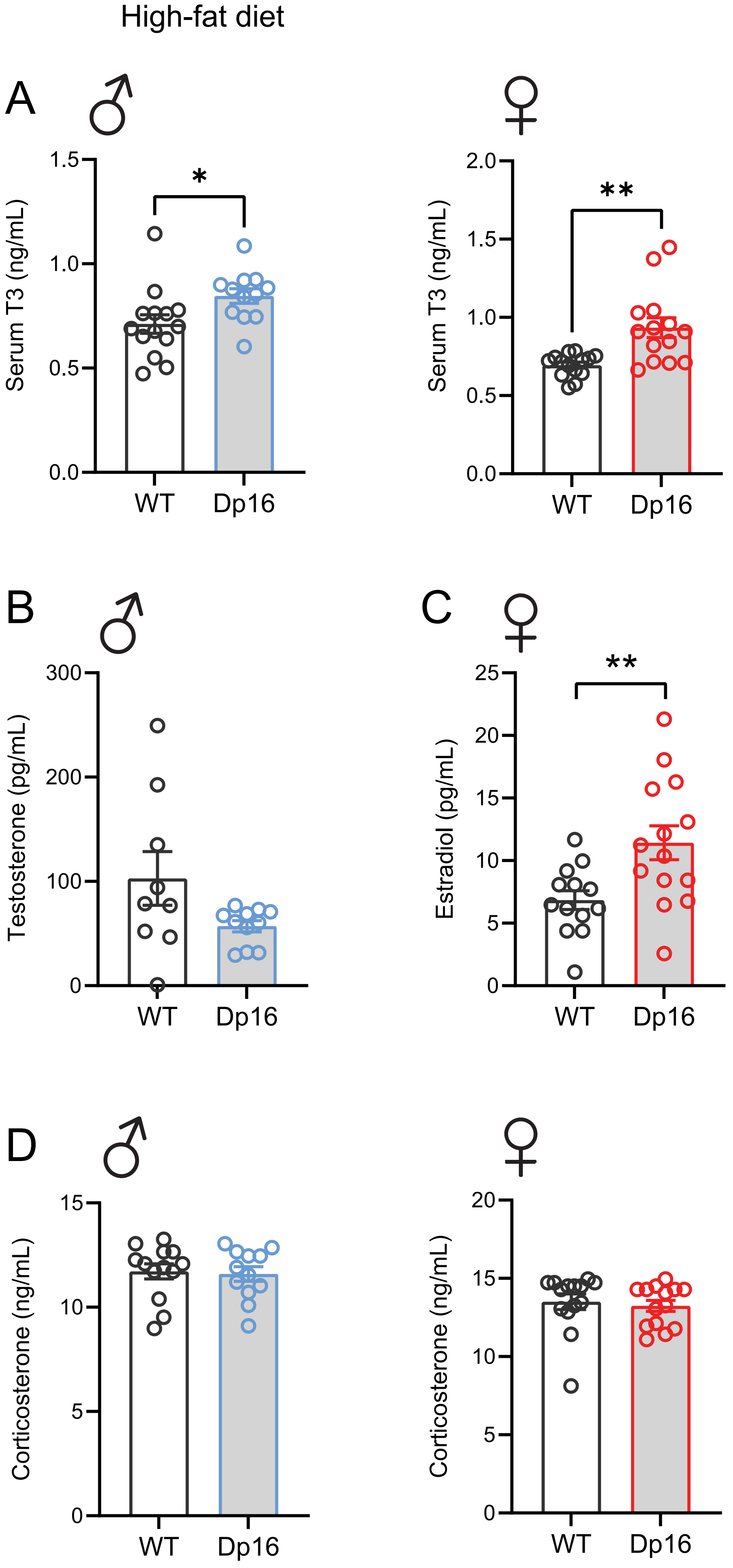

bioRxiv preprint doi: https://doi.org/10.64898/2026.01.13.699318; this version posted January 14, 2026. The copyright holder for this preprint (which was not certified by peer review) is the author/funder, who has granted bioRxiv a license to display the preprint in perpetuity. It is made available under aCC-BY 4.0 International license.

Fig. 6 - figure supplement 3

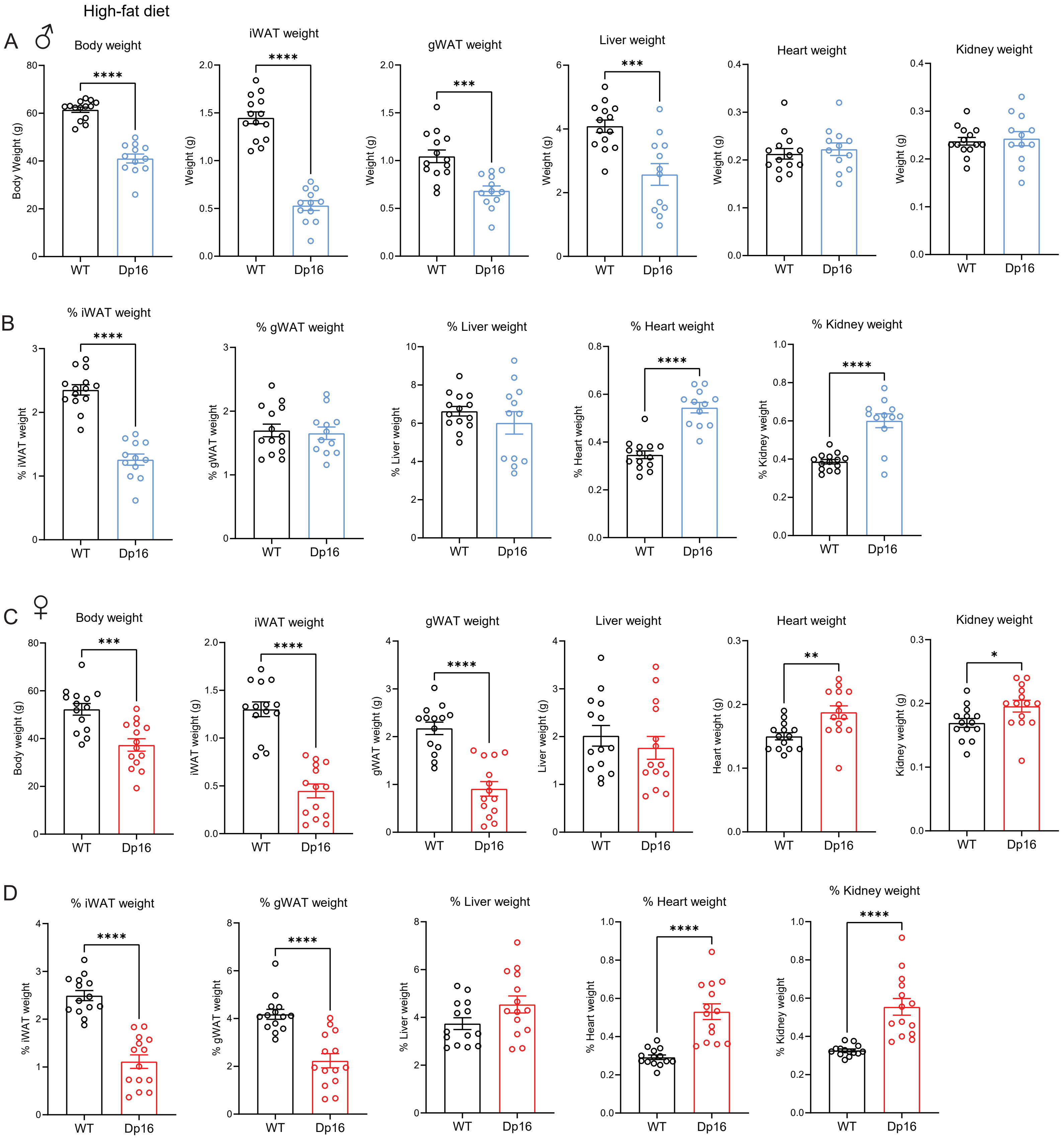

bioRxiv preprint doi: https://doi.org/10.64898/2026.01.13.699318; this version posted January 14, 2026. The copyright holder for this preprint (which was not certified by peer review) is the author/funder, who has granted bioRxiv a license to display the preprint in perpetuity. It is made available under aCC-BY 4.0 International license.

Fig 6- figure supplement 4

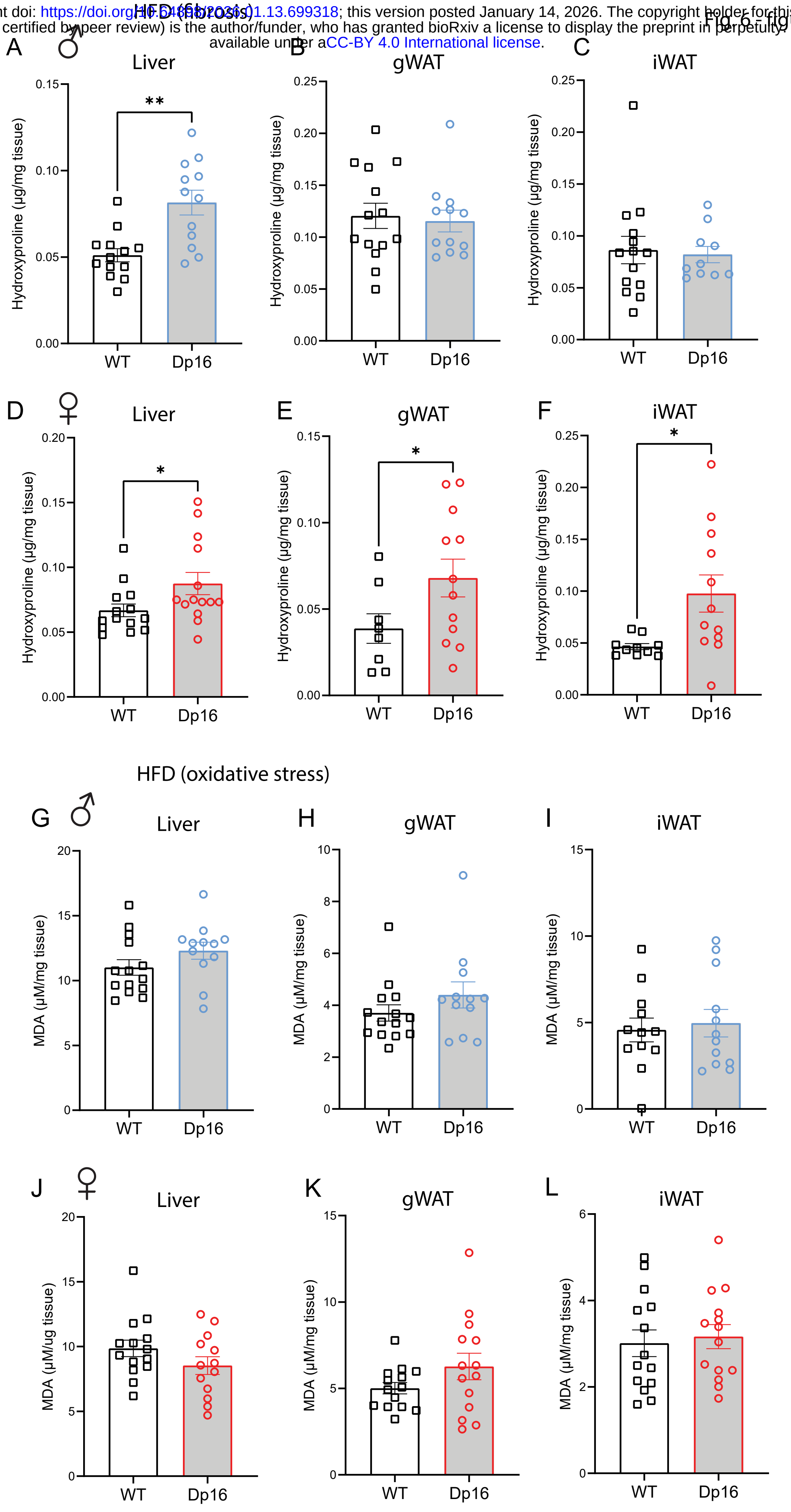

Supplement: Supplement 1 [file NIHPP2026.01.13.699318v2-supplement-1.pdf]
